# Comparison of Rates and Outcomes of Readmission to Index vs Non-index Hospitals After Intravenous Thrombolysis in Acute Stroke Patients

**DOI:** 10.7759/cureus.8952

**Published:** 2020-07-01

**Authors:** Kristina Shkirkova, Michelle Connor, Drew M Hodis, Krista Lamorie-Foote, Arati Patel, Qinghai Liu, Li Ding, Arun Amar, Nerses Sanossian, Frank Attenello, William Mack

**Affiliations:** 1 Neurological Surgery, University of Southern California, Keck School of Medicine, Los Angeles, USA; 2 Neurological Surgery, University of Southern California, Los Angeles, USA; 3 Zilkha Neurogenetic Institute, University of Southern California, Los Angeles, USA; 4 Neurological Surgery, University of California, San Francisco, San Francisco, USA; 5 Preventive Medicine, University of Southern California, Los Angeles, USA; 6 Neurology, University of Southern California, Los Angeles, USA

**Keywords:** stroke, thrombolysis, readmission, stroke systems of care

## Abstract

National and regional systems of stroke care are designed to provide patients with widespread access to hospitals with thrombolytic capabilities. However, such triaging systems may contribute to fragmentation of care. This study aims to compare rates of readmission and outcomes between index and non-index hospitals for stroke patients following intravenous thrombolytic therapy (IVT). This study utilized a nationally representative sample of stroke patients with IVT from the Nationwide Readmissions Database from 2010 to 2014. Descriptive and regression analyses were performed for patient and hospital level factors that influenced 90-day readmissions and regression models were used to identify differences in mortality, complications, and repeat readmissions between patients readmitted to index (facility where IVT was administered) and non-index hospitals. In the study, 49415 stroke patients were treated with IVT, of whom 21.7% were readmitted within 90 days. Among readmissions, 79.4% of patients were readmitted to index hospitals and 20.6% to non-index hospitals. On multivariate logistic regression analysis, index hospital readmission was independently associated with lower frequency of second readmissions (non-index OR 1.09, 95%CI 1.07-1.11, p<0.0001) but not with increased mortality or major complications (p=ns). Approximately one-fifth of stroke patients treated with thrombolysis were readmitted within 90 days, one-fifth of whom were readmitted to non-index hospitals. Although readmission to index hospital was associated with lower frequency of subsequent readmissions, readmission to non-index hospital was not associated with increased mortality or major complications. This difference may be due to standardized algorithms, mature systems of care, and demanding metrics required of stroke centers.

## Introduction

An estimated 795,000 people suffer from stroke in the United States each year [1]. Eighty-seven percent of these strokes are ischemic. Management of ischemic stroke is time-sensitive as clinical symptoms often progress rapidly to permanent neurological deficits. Pre-hospital neurological deterioration occurs in 9% of ischemic stroke patients [2]. Roughly 7% of patients deteriorate during the early Emergency Department (ED) phase [2], and approximately 20% experience neurological worsening within the first 24 to 48 hours at the hospital [3]. Vessel recanalization and restoration of perfusion to viable brain tissue is the most effective means of limiting ischemic stroke progression. Originally approved in 1996 for use in the United States after the positive National Institute of Neurological Disorders and Stroke (NINDS) trial [4], thrombolysis with intravenous tissue plasminogen activator (IV-tPA) is considered to be the standard of care for eligible patients suffering from acute ischemic stroke [4]. The recommended window for IV-tPA treatment is within 3, or in some cases 4.5, hours after the onset of stoke [5, 6]. It is, therefore, critical for ischemic stroke patients to be brought to a capable stroke center in rapid fashion following onset of symptoms.

National and regional systems of stroke care aim to provide patients with widespread access to hospitals with thrombolytic capabilities, as early intervention with IV-tPA is critical for improved outcomes. Emergency Medical Services (EMS) protocols dictate routing of suspected stroke patients to certified stroke hospitals such as Primary Stroke Centers (PSCs) to expedite in-hospital time-to-treatment processes. However, ischemic stroke patients treated with IV-tPA have risks of disease- and treatment- related complications, as well as comorbidities, that typically require specialized in-hospital and follow-up management [1, 7, 8]. Stroke systems and routing parameters often result in patients initially being treated in hospitals that are further from their homes. This may negatively impact continuity of care and management of complications in cases of readmission (potentially to a different hospital) [9].

In a number of medical conditions, readmission to a different hospital is associated with less favorable outcomes due to care fragmentation [10, 11]. Our study leverages the Nationwide Readmission Database to determine rates of readmission to index (site of first admission) and non-index (site other than first admission) hospitals for stroke patients following thrombolysis therapy and to compare readmission outcomes between the index and non-index readmission types.

## Materials and methods

Data Source

The Healthcare Cost and Utilization Project Nationwide Readmissions Database (NRD) is a nationally representative database with information about patients with readmissions following initial hospitalization. The NRD database collects data from 28 states, representing about 58% of all hospitalizations and readmissions in the US. The NRD provides a unique identifier link for each patient, within each calendar year, within each state, that is used to determine patients’ hospitalizations within that year. The index hospital was defined as the hospital at which the patient was initially admitted and treated with IVT, while a non-index hospital was defined as any other hospital. The NRD is a de-identified, publicly available database. No institutional review board or ethics approval was required for this study. This study is a retrospective analysis of the NRD database from 2010-2014.

Inclusion and Exclusion Criteria

International Classification of Disease, Ninth Edition, Clinical Modifications (ICD-9-CM) diagnosis and procedure codes were used to identify patients meeting inclusion criteria. Adult patients (≥18 years old) with a diagnosis of ischemic stroke (433.01, 433.11, 433.21, 433.31, 433.81, 433.91, 434.01, 434.11, 434.91, 436) managed with IVT (99.10, V45.88) were selected from the NRD and included in this study. Only non-elective admissions were considered in this study. As the NRD records patient data for a single calendar year, only patients with sufficient follow-up time of 90 days (discharged between January and September) were included in this study. Only the first readmission within 90-days was included in this analysis. However, rates of repeat readmission were measured.

Patients with a diagnosis of head trauma (80x.xx, 85x.xx), subarachnoid hemorrhage (430), extra-axial hematoma (432.0, 432.1, 432.9), arteriovenous malformation (747.81), cerebral arteritis (437.4), Moyamoya disease (437.5), venous sinus thrombosis (437.6), brain tumor (191.x, 192.0, 192.1, 194.3, 198.3, 198.4, 199.0, 200.5, 225.x, 227.3, 237.0, 237.5, 237.6), or intracranial abscess (324.0) were excluded. Additionally, patients who underwent microsurgical clipping of a cerebral aneurysm (39.51, 39.52), repair of an arteriovenous malformation (39.53), cranioplasty (02.03-02.07), stereotactic radiosurgery (92.3x), carotid artery stent placement (00.63), or carotid endarterectomy (38.12) were excluded. Patients who died during the index hospitalization, or who were missing “died” or “length of stay” information were excluded. Patients identified according to the above inclusion and exclusion criteria were included as study participants.

Association Variables

Patient and hospital level characteristics were included in the analysis. Patient demographics included age (categorized as 18-44, 45-59, 60-74, >75), gender (male or female), insurance type (Medicare, Medicaid, private, self-pay, no charge, or other), median household income (by quartile), residency status (resident/nonresident of state where procedure was performed), discharge quarter (January-March, April-June, July-September), and discharge disposition (routine vs other). Other patient characteristics included comorbidity score, major complications, neurological complications, and second readmission. Comorbidity score was determined by the Elixhauser variables and re-categorized into 3 groups (0-1, 2, or ≥3). Major complications included pneumonia (481-482, 482.1-482.3, 482.30-482.32, 482.39-482.41, 482.49, 482.80-482.84, 482.89, 482.90, 483.0, 483.1, 483.8, 485-487.0, 997.3, 507.0), pulmonary embolism (415.1-415.9), renal failure (584, 584.5-584.9), cerebrovascular accident (433.01, 433.11, 433.21, 433.31, 433.81, 433.91), myocardial infarction (410.00-410.90, 410.01, 410.11-410.91), cardiac arrest (427.5), sepsis (995.91), and septic shock (995.92). Neurological complications included intracerebral hemorrhage (431, 998.11-998.12), seizures (345.xx), and neurological complications after procedure (997.01-997.09). All patient-refined diagnosis related groups (APR-DRG) severity of illness and risk of mortality scores (minor, moderate, major, extreme) provided by NRD were also included. Hospital characteristics included bed size (small, medium, or large), academic vs non-academic status, ownership status (government, nonfederal; private, nonprofit; private, invest-own), and urban vs rural location.

Outcome Variables

Rates of readmission to index or non-index hospitals within 90 days of initial hospitalization were quantified. Reasons for readmission, based on ICD-9-CM codes, were identified. Patient and hospital factors associated with readmission were evaluated. Outcomes following readmission to index or non-index hospitals, including mortality, major complications, neurological complications, and second readmission were assessed. Factors associated with mortality, major complications, neurological complications, and second readmission were identified.

Statistical Analysis

Descriptive analyses were performed to characterize patient and hospital factors associated with 90-day readmission to index or non-index hospitals. Multivariable logistic regression models were used for mortality, major complications, neurological complications, and frequency of second readmissions. Patient and hospital level demographic variables from the initial hospitalization (listed above) were included in the models. A sensitivity analysis was performed to adjust for readmission hospital characteristics. Data was reported using odds ratios (OR) and 95% confidence intervals (CI). Statistical significance was defined as p<0.05. Statistical analysis was conducted using SAS 9.4 (SAS Inc., Cary, North Carolina, USA).

## Results

Study Participants and Descriptive Data

A total of 49415 patients with ischemic stroke were treated with IV-tPA during the study period. Among them, 10718 (21.7%) patients were readmitted within 90 days, which constituted the primary population of this study. Of the 10718 readmitted patients, 8514 (79.4%) were readmitted to the index hospital and 2204 (20.6%) were readmitted to a non-index hospital. Age distribution of the study population was: 18-44 (4.8%), 45-59 (17.8%), 60-74 (32.5%), ≥75 (44.8%). Percentage of female patients was 49.9%. (Table 1).

**Table 1:** Patient and Hospital Characteristics at Index and Non-index 90-Day Readmissions * for patient's ZIP code, based on current year DS* – data with less than 10 patients is suppressed

	Total (n=10718)	Index Hospital (n=8514)	Non-index Hospital (n=2204)	p-value
Age, n (%)				
18-44	517 (4.8)	387 (4.6)	130 (5.9)	0.0007
45-59	1910 (17.8)	1489 (17.5)	421 (19.1)	
60-74	3486 (32.5)	2747 (32.3)	739 (33.5)	
≥75	4805 (44.8)	3891 (45.7)	914 (41.5)	
Female, n (%)	5349 (49.9)	4263 (50.1)	1086 (49.3)	0.51
Primary Insurance, n (%)				
Medicare	7357 (68.8)	5899 (69.3)	1476 (67.0)	0.0071
Medicaid	977 (9.1)	733 (8.6)	244 (11.1)	
Private insurance	1711 (16.0)	1365 (16.0)	346 (15.7)	
Self-pay	358 (3.3)	278 (3.3)	80 (3.6)	
No charge	43 (0.4)	38 (0.5)	DS* (0.2)	
Other	234 (2.2)	186 (2.2)	48 (2.2)	
Missing	20 (0.2)	15 (0.2)	DS* (0.2)	
Comorbidity Score, n (%)				
0	209 (1.9)	162 (1.9)	47 (2.1)	0.22
1	995 (9.3)	782 (9.2)	213 (9.7)	
2	1868 (17.4)	1515 (17.8)	353 (16.0)	
3	7646 (71.3)	6055 (71.1)	1591 (72.2)	
Median Household Income*, n (%)				
0-25 percentile	2961 (27.6)	2304 (27.1)	657 (29.8)	0.025
26-50 percentile	2568 (24.0)	2027 (23.8)	541 (24.6)	
51-75 percentile	2585 (24.1)	2072 (24.3)	513 (23.3)	
76-100 percentile	2445 (22.8)	1979 (23.2)	466 (21.1)	
Missing	159 (1.5)	132 (1.6)	27 (1.2)	
All Patient Refined DRG: Risk of Mortality, n (%)				
Minor	2324 (21.7)	1827 (21.5)	497 (22.6)	0.0155
Moderate	4085 (38.1)	3293 (38.7)	792 (35.9)	
Major	2485 (23.2)	1986 (23.3)	499 (22.6)	
Extreme	1824 (17.0)	1408 (16.5)	416 (18.9)	
All Patient Refined DRG: Severity of Illness Subclass, n (%)				
Minor	465 (4.3)	365 (4.3)	100 (4.5)	0.11
Moderate	4114 (38.4)	3299 (38.8)	815 (37.0)	
Major	4245 (39.6)	3382 (39.7)	863 (39.2)	
Extreme	1894 (17.7)	1468 (17.2)	426 (19.3)	
Control/Ownership of Hospital, n (%)				
Government, non-federal	120 (22.9)	87 (22.5)	33 (24.2)	0.0123
Private, non-profit	7648 (71.4)	6126 (72.0)	1522 (69.1)	
Private, invest-own	1509 (14.1)	1188 (14.0)	321 (14.6)	
Teaching Status of Urban Hospitals, n (%)				
Teaching	6608 (61.7)	5198 (61.1)	1410 (64.0)	0.0119
Non-teaching	4110 (38.3)	3316 (39.0)	794 (36.0)	
Hospital Bed Size, n (%)				
Small	629 (5.9)	474 (5.6)	155 (7.0)	0.0331
Medium	2327 (21.7)	1856 (21.8)	471 (21.4)	
Large	7762 (72.4)	6184 (72.6)	1578 (71.6)	
Hospital Urban-Rural Designation, n (%)				
Large metropolitan areas with at least 1 million residents, n (%)	6583 (61.4)	5134 (60.3)	1449 (65.7)	<0.0001
Other	4135 (38.6)	3380 (39.7)	755 (34.3)	
Resident of state where procedure was performed, n (%)				
Nonresident	334 (3.1)	276 (3.2)	58 (2.6)	0.14
Resident	10384 (96.9)	8238 (96.8)	2146 (97.4)	
Discharged to Another Facility, n (%)				
Yes	7475 (69.7)	5900 (69.3)	1575 (71.5)	0.0417
No	3237 (30.2)	2611 (30.7)	626 (28.4)	
Major Complication, n (%)				
Yes	3174 (29.6)	2514 (29.5)	660 (30.0)	0.70
No	7544 (70.4)	6000 (70.5)	1544 (70.1)	
Neurological Complication, n (%)				
Yes	1300 (12.1)	993 (11.7)	307 (13.9)	0.0037
No	9418 (87.9)	7521 (88.3)	1897 (86.1)	
Repeat Readmission, n (%)				
Yes	2572 (24.0)	1882 (22.1)	690 (31.3)	<0.0001
No	8146 (76.0)	6632 (77.9)	1514 (68.7)	
Discharge Quarter, n (%)				
Jan-March	3501 (32.7)	2773 (32.6)	728 (33.0)	0.79
April-June	3562 (33.2)	2843 (33.4)	719 (32.6)	
July-Sep	3655 (34.1)	2898 (34.0)	757 (34.4)	

The most common reasons for readmission were cerebral artery occlusion (8.73%), septicemia (5.83%), and carotid artery occlusion (5.22%) (Table 2).

**Table 2:** Most frequent reasons for 90-day readmission

ICD	Diagnosis	n (%)
43491	Cerebral Artery Occlusion w/ Infarct	935 (8.7)
0389	Septicemia	624 (5.8)
43310	Carotid Artery Occlusion w/out Infarct	559 (5.2)
4359	Transient Cerebral Ischemia	311 (2.9)
5990	Urinary Tract Infection	308 (2.9)
42731	Atrial Fibrillation	267 (2.5)
5070	Food/Vomit Pneumonitis	257 (2.4)
43411	Cerebral Embolism w/ Infarction	205 (1.9)
V5789	Rehabilitation Procedure	204 (1.9)
486	Pneumonia	195 (1.8)

Readmission to Index or Non-Index Hospitals

In the multivariable logistic regression model of index vs non-index readmission, older age (60-74 years old: OR 0.78, 95% CI 0.63-0.97, p=0.03; ≥75 years old: OR 0.66, 95% CI 0.53-0.83, p=0.0003) and higher median household income (51-75 percentile: OR 0.85, 95% CI 0.74-0.98, p=0.02; 76-100 percentile: OR 0.76, 95% CI 0.66-0.88, p=0.0003) were associated with readmission to index hospital. Non-routine discharge (OR 1.19, 95% CI 1.06-1.33, p=0.002) and neurological complication (OR 1.19, 95% CI 1.03-1.36, p=0.01) were associated with readmission to a non-index hospital. Among hospital level factors, hospital location in a rural area (OR 0.77, 95% CI 0.69-0.86, p<0.0001) was associated with readmission to index hospital, while small hospital bed size (OR 1.34, 95% CI 1.11-1.62, p=0.003) was associated with readmission to a non-index hospital.

Outcomes following Index vs Non-Index Hospital Readmission

Readmission to non-index hospital was associated with increased frequency of repeat readmissions (OR 1.09, 95% CI 1.06-1.11, p<0.0001). Readmission to a non-index hospital was not associated with mortality (OR 1.00, 95% CI 0.99-1.01, p=0.44), major complications (OR 1.00, 95% CI 0.98-1.02, p=0.84), or neurological complications (OR 1.01, 95% CI 0.99-1.02, p=0.32) (Table 3).

**Table 3:** Summary Readmission to Index vs Non-index Hospitals Associations The multivariate model is additionally adjusted for age, sex, insurance status, discharge quarter, hospital volume, risk of mortality, disease severity, major complications during hospital stay, length of stay, and hospital ownership

Outcome	Index Hospital	Non-index Hospital	OR (95%CI)	p-value
Mortality	Reference	1.00	0.99-1.01	0.44
Major Complication	Reference	1.00	0.98-1.02	0.84
Neurological Complication	Reference	1.01	0.99-1.02	0.32
Repeat Readmission	Reference	1.09	1.07-1.11	<0.0001

Outcomes following Readmission in Overall Index and Non-Index Group

Patient and hospital factors associated with mortality, major complications, neurological complications, and repeat readmission were assessed. Patient factors associated with mortality included increased age, higher risk of mortality defined as an APR-DRG classification of major or extreme, neurological complication, and discharge to another facility (Table 4).

**Table 4:** Summary of Associations with Mortality The multivariate model is additionally adjusted for age, sex, insurance status, discharge quarter, hospital volume, risk of mortality, disease severity, major complications during hospital stay, length of stay, and hospital ownership

	OR	95% CI	p-value
Hospital			
Index hospital	Ref		
Non-index hospital	1.00	0.99-1.01	0.44
Age			
18-44	Ref		
45-59	1.01	1.00-1.02	0.02
60-74	1.02	1.01-1.03	<0.0001
≥ 75	1.05	1.03-1.06	<0.0001
All Patient Refined DRG: Risk of Mortality			
Minor	Ref		
Moderate	1.00	0.99-1.01	0.81
Major	1.04	1.03-1.05	<0.0001
Extreme	1.17	1.15-1.20	<0.0001
Discharged to Another Facility			
Yes	1.02	1.01-1.03	<0.0001
No	Ref		
Neurological Complication			
Yes	0.98	0.96-0.99	0.001
No	Ref		

Increased age, male gender, greater illness severity (APR-DRG of moderate, major, extreme), and presence of a major complication at initial hospitalization were associated with major complications. Presence of a neurological complication at initial hospitalization and shorter length of stay were associated with a lower rate of major complications (Table 5).

**Table 5:** Summary of Associations with Major Complication The multivariate model is additionally adjusted for age, sex, insurance status, discharge quarter, hospital volume, risk of mortality, disease severity, major complications during hospital stay, length of stay, and hospital ownership

	OR	95% CI	p-value
Hospital			
Index hospital	Ref		
Non-index hospital	1.00	0.98-1.02	0.84
Age			
18-44	Ref		
45-59	1.01	0.98-1.05	0.40
60-74	1.07	1.04-1.10	<0.0001
≥ 75	1.13	1.10-1.17	<0.0001
Gender			
Male	1.04	1.03-1.06	<0.0001
Female	Ref		
All Patient Refined DRG: Disease Severity			
Minor	Ref		
Moderate	1.07	1.05-1.09	<0.0001
Major	1.30	1.27-1.33	<0.0001
Extreme	1.70	1.65-1.76	<0.0001
Major Complication			
Yes	1.08	1.06-1.10	<0.0001
No	Ref		
Neurological Complication			
Yes	0.96	0.94-0.98	0.001
No	Ref		
Length of Stay			
Per 1 day increase	1.00	1.00-1.00	0.0003

Illness severity (APR-DRG of moderate, major, extreme), neurological complication at initial hospitalization, and discharge to another facility were associated with higher rate of neurological complication at readmission (Table 6).

**Table 6:** Summary of Associations with Neurological Complication The multivariate model is additionally adjusted for age, sex, insurance status, discharge quarter, hospital volume, risk of mortality, disease severity, major complications during hospital stay, length of stay, and hospital ownership

	OR	95% CI	p-value
Hospital			
Index hospital	Ref		
Non-index hospital	1.01	0.99-1.02	0.32
Age			
18-44	Ref		
45-59	1.00	0.97-1.03	0.96
60-74	0.98	0.95-1.01	0.18
≥ 75	0.95	0.93-0.98	0.0002
All Patient Refined DRG: Disease Severity			
Minor	Ref		
Moderate	1.04	1.02-1.05	<0.0001
Major	1.06	1.04-1.07	<0.0001
Extreme	1.08	1.06-1.11	<0.0001
Major Complication			
Yes	0.98	0.97-0.99	0.002
No	Ref		
Neurological Complication			
Yes	1.24	1.21-1.28	<0.0001
No	Ref		
Discharged to Another Facility			
Yes	1.02	1.00-1.03	0.011
No	Ref		

Repeat readmission was associated with comorbidity score >3, APR-DRG disease severity score of major, and longer length of stay. Older age, private insurance, household income in the 76-100 percentile, being a non-resident of the state where the procedure was performed, and rural hospital location were associated with lower frequency of repeat readmission (Table 7).

**Table 7:** Summary of Associations with Repeat Readmission The multivariate model is additionally adjusted for age, sex, insurance status, discharge quarter, hospital volume, risk of mortality, disease severity, major complications during hospital stay, length of stay, and hospital ownership

	OR	95% CI	p-value
Hospital			
Index hospital	Ref		
Non-index hospital	1.09	1.07-1.11	<0.0001
Age			
18-44	Ref		
45-59	0.99	0.95-1.03	0.60
60-74	0.96	0.91-1.00	0.04
≥ 75	0.93	0.89-0.98	0.002
Primary Insurance			
Medicaid	Ref		
Medicare	1.00	0.97-1.04	1.00
Private insurance	0.95	0.92-0.99	0.005
Self-pay	0.97	0.92-1.02	0.21
No charge	0.99	0.87-1.13	0.89
Other	0.94	0.89-1.00	0.06
Comorbidity Score			
0	Ref		
1	1.01	0.95-1.06	0.81
2	1.04	0.99-1.09	0.16
≥ 3	1.09	1.04-1.15	0.0009
Median Household Income			
0-25 percentile	Ref		
26-50 percentile	0.98	0.96-1.00	0.10
51-75 percentile	0.98	0.96-1.00	0.11
76-100 percentile	0.96	0.94-0.98	0.001
All Patient Refined DRG: Disease Severity			
Minor	Ref		
Moderate	1.02	0.99-1.05	0.13
Major	1.04	1.01-1.08	0.007
Extreme	1.02	0.99-1.06	0.25
Hospital urban-rural designation			
Large metropolitan area with at least 1 million residents	Ref		
Other	0.97	0.96-0.99	0.002
Resident of state where procedure was performed			
Nonresident	0.93	0.89-0.97	0.0008
Resident	Ref		
Length of Stay			
Per 1 day increase	1.00	1.00-1.00	0.005

Sensitivity analysis adjusting for characteristics of readmission hospitals was performed, and outcome results were the same.

## Discussion

Ninety-day hospital readmission rates are important patient safety indicators and potential drivers of quality improvement initiatives. Understanding of readmission incidences, demographics, and outcomes represents an opportunity to identify patients at risk for complications and opportunities for improved systems of care. In our study, one-fifth (21.7%) of acute ischemic stroke (AIS) patients treated with IV-tPA were readmitted within 90 days of initial hospitalization, of which 20.6% were readmissions to non-index hospitals. Prior ischemic stroke studies have reported 7-12% 30-day readmission rates [12-17]. A large ischemic stroke hospital registry documented readmission rates of 10% at 30 days, 17% at 90 days, 24% at 180 days, and 36% at 360 days [18]. IV-tPA treatment is clearly beneficial to properly selected acute stroke patients. However, its administration is associated with risk of post-treatment complications such as intracranial hemorrhage, angioedema, re-occlusion, secondary embolization, and neurotoxicity [19-22]. Recurrent strokes and seizures may also occur following TPA administration as a function of the initial stroke etiology or a complication related to the ischemic tissue bed [23, 24]. The most common cause for readmission within 30 days not related to IV-tPA is acute cerebrovascular disease, accounting for nearly 20% of readmitted patients [12, 17]. Previous nationwide studies in stroke suggest that infection is the second leading cause of 30-day readmission (10-15% of patients) [12, 14]. Our results demonstrate a similar pattern, with cerebral artery occlusion (8.73%), septicemia (5.83%), and carotid artery occlusion (5.22%) as the most common causes for readmission within 90 days. This suggests that while cerebrovascular disease may account for a large proportion of early readmissions, other underlying disease processes or complications may be important in disease progression.

Fragmentation of care and readmission to a non-index hospital has been associated with higher rates of morbidity and mortality in other disease processes [11, 25]. While our results indicate that readmission to a non-index hospital after IVT for AIS was associated with more frequent secondary readmissions, it did not result in worse outcomes. Overall, patients readmitted to index vs. non-index hospitals did not differ with regards to mortality, major complications, or neurological complications. This may be due to mature systems of stroke care and standardization of IV-tPA administration. Patients suspected of having a stroke are typically directed to a hospital that is capable of administering IV-tPA. These hospitals include acute stroke ready hospitals (ASRH) and primary stroke centers (PSC) that are uniquely equipped to diagnose and treat patients with an ischemic stroke. ASRH are responsible for stabilizing the patient, providing initial therapy, and arranging efficient transport to a higher level of care. PSCs are capable of managing the majority of acute stroke patients and have designated care teams, stroke units, protocols, and rapid imaging and laboratory services [26, 27]. The methods and procedures for IV-tPA infusion and patient monitoring/ management are standardized and very well regulated according to guidelines. While there is likely variation to other procedures and ancillary services that an IV-tPA- treated stroke patient may have received, there is little ambiguity as to the thrombolysis treatment and related protocols at the time of initial stroke hospitalization. This makes it easier for medical teams to administer care and necessary treatments upon readmission. There may be less need for detailed history of the initial IV-tPA treatment, protocols, blood pressure parameters, and imaging paradigms. This is not true for many other disease processes where surgeries, chemotherapy regimens, or treatment protocols could vary greatly across disease types/ progression and different hospital systems. Standardization of acute stroke care across healthcare providers results in more comprehensive and coherent care of patients with ischemic stroke. The present study suggests that stroke systems of care are effective in the follow-up period, regardless of readmission destination.

This study has limitations. This study relies on retrospective data from the Nationwide Readmissions Database. Data is therefore subject to coding errors and information bias. The NRD includes only readmissions occurring in the same state as initial treatment and out-of-state readmissions are unable to be included in the analysis. There are a limited number of variables available in the NRD. Notably, race and ethnicity are not included, so the effect of these factors cannot be determined. Despite these limitations inherent to the database, the large sample size and use of multivariable analysis allows us to assess outcomes of 90-day readmissions.

## Conclusions

Readmission to non-index hospital following IV-tPA treatment for acute stroke was not associated with increased mortality or major complications, unlike what has been seen in prior studies of other diseases processes. This difference may be due to standardized algorithms, mature systems of care, and demanding metrics required of acute stroke centers.
